# Metabolomic Analysis of Patients with Chronic Myeloid Leukemia and Cardiovascular Adverse Events after Treatment with Tyrosine Kinase Inhibitors

**DOI:** 10.3390/jcm9041180

**Published:** 2020-04-20

**Authors:** Giovanni Caocci, Martino Deidda, Antonio Noto, Marianna Greco, Maria Pina Simula, Olga Mulas, Daniele Cocco, Claudia Fattuoni, Giuseppe Mercuro, Giorgio La Nasa, Christian Cadeddu Dessalvi

**Affiliations:** 1 SC Ematologia e CTMO Dipartimento di Scienze Mediche e Sanità Pubblica, P.O. Businco, Azienda Ospedaliera Brotzu, Università di Cagliari, 09134 Cagliari, Italy; giovanni.caocci@unica.it; 2 Department of Medical Sciences and Public Health, University of Cagliari, 09042 Cagliari, Italy; antonionoto@unica.it (A.N.); daniele.cocco89@gmail.com (D.C.); giuseppemercuro@gmail.com (G.M.); cadedduc@unica.it (C.C.D.); 3 Anatomia Patologica, P.O. Businco, Azienda Ospedaliera Brotzu, 09134 Cagliari, Italy; mgreco71@gmail.com; 4 SC Ematologia e CTMO P.O. Businco, Azienda Ospedaliera Brotzu, 09121 Cagliari, Italy; mpinasimula@hotmail.com (M.P.S.); mulasolga@gmail.com (O.M.); lanasa@tiscali.it (G.L.N.); 5 Department of Chemical of Chemical and Geological Sciences, University of Cagliari, 09042 Cagliari, Italy; cfattuon@unica.it

**Keywords:** Chronic myeloid leukemia, cardiovascular adverse events, tyrosine kinase inhibitors, GC-MS metabolomics

## Abstract

Background: Cardiovascular adverse events (CV-AEs) are considered critical complications in chronic myeloid leukemia (CML) patients treated with second- and third-generation tyrosine kinase inhibitors (TKIs). The aim of our study was to assess the correlation between metabolic profiles and CV-AEs in CML patients treated with TKIs. Methods: We investigated 39 adult CML patients in chronic-phase (mean age 49 years, range 24–70 years), with no comorbidities evidenced at baseline, who were consecutively identified with CML and treated with imatinib, nilotinib, dasatinib, and ponatinib. All patients performed Gas-Chromatography-Mass-Spectrometry-based metabolomic analysis and were divided into two groups (with and without CV-AEs). Results: Ten CV-AEs were documented. Seven CV-AEs were rated as 3 according to the Common Toxicity Criteria, and one patient died of a dissecting aneurysm of the aorta. The patients’ samples were clearly separated into two groups after analysis and the main discriminant metabolites were tyrosine, lysine, glutamic acid, ornithine, 2-piperdinecarboxylic acid, citric acid, proline, phenylalanine, threonine, mannitol, leucine, serine, creatine, alanine, and 4-hydroxyproline, which were more abundant in the CV-AE group. Conversely, myristic acid, oxalic acid, arabitol, 4-deoxy rithronic acid, ribose, and elaidic acid were less represented in the CV-AE group. Conclusions: CML patients with CV-AEs show a different metabolic profile, suggesting probable mechanisms of endothelial damage.

## 1. Introduction

Chronic myeloid leukemia (CML) is a myeloproliferative disease characterized by a chromosomal translocation that leads to the constitutive activation of tyrosine kinase (BCR-ABL1) [1]. CML can be fatal if left untreated, and its treatment has definitely changed with the introduction of tyrosine kinase inhibitors (TKIs), which specifically target the BCR-ABL1 oncoprotein [1]. Since the year 2000, the survival rate has been progressively increasing and the life expectancy of patients with CML is reaching that of the general population [2]. Nevertheless, all TKIs have a broad spectrum of off-target toxicity, including endocrine disorders and cardiovascular adverse events (CV-AEs) [3,4], which represent emerging complications outside clinical trials. Imatinib, the first TKI, has a relatively low cardiotoxicity, whereas second- and third-generation TKIs (dasatinib, nilotinib, bosutinib, and ponatinib) have been reported to have multiple vascular adverse effects, including vascular occlusive events, peripheral arterial occlusive disease, QT interval prolongation, and pulmonary hypertension [5,6,7]. Accumulating evidence suggests that the combination of a diagnosis of CML in patients aged > 60 years, in whom CV-AEs are common, and the endothelial toxicity of TKIs represents a predisposing factor that requires careful cardiovascular (CV) surveillance [7]. Despite the importance of CV risk factors and/or a history of CV disease in identifying CML patients with a risk of developing CV-AEs, predictive biomarkers are still lacking. Laboratory studies have already provided insights into the molecular mechanisms associated with the pathophysiology of CML [8]. However, the development of CV-AEs in CML patients is still not well understood. A comprehension of the molecular pathways underlying CV-AEs may suggest new strategies to prevent initiation and progression to clinical disease. In this scenario, metabolomics may be useful for the characterization of metabolic pathways related to CV-AEs [9]. Indeed, metabolomics represent an innovative technology in medicine, potentially able to translate the metabolic fingerprint into personalized treatment strategies [10,11]. Metabolomics helps in detection, identification, and quantification of a large number of metabolites in a biological sample. Metabolites represent the fingerprint of cellular metabolism, expression and activity of genes, transcripts, and proteins, and offer further insights into small-molecule regulation. Oxidative stress plays a major role in endothelial toxicity leading to CV-AEs, and the metabolomic approach can be used to understand the mechanism underlying this damaging process [9]. 

To our best knowledge, only a few papers have considered the role of metabolomics in onco-hematological diseases [12,13,14,15] and no study has evaluated a possible application of metabolomics in CV toxicity after treatment with TKIs. Therefore, in this exploratory study, we evaluated the potential correlation between metabolic profiles and CV-AEs onset in a monocentric cohort of CML patients treated with TKIs.

## 2. Experimental Section

### 2.1. Study Population

We analyzed 39 adult chronic-phase CML patients (mean age 49 years, range 24–70 years), without comorbidities at baseline, who were consecutively diagnosed and treated with imatinib, dasatinib, nilotinib, and ponatinib at the Haematology Unit of Businco Hospital, Cagliari, Italy. CV-AEs were recorded during TKI treatment. A review of medical charts allowed us to retrospectively collect information on CV risk factors. To estimate the Systematic Coronary Risk Evaluation (SCORE) risk, a 10-year risk estimation of fatal CV disease based on age, sex, smoking habits, systolic pressure, and total cholesterol level [16], all patients were evaluated at diagnosis for these variables and stratified into two classes of CV Risk: low–moderate (SCORE ≤ 5%) or high–very high (SCORE > 5%). Patients were also evaluated for comorbidities (diabetes, body mass index (BMI) > 24.9 kg/m^2^, mild or severe renal insufficiency) and a positive anamnesis of CV diseases, including angina, myocardial infarction, arterial hypertension, heart failure, cardiomyopathy, heart arrhythmia, valvular heart disease, stroke, ischemic cerebrovascular events, peripheral artery disease, aortic aneurysms, thromboembolic disease, and venous thrombosis. Ongoing primary or secondary antithrombotic prophylaxis was also evaluated. Therefore, we collected information on any CV-AE which occurred after any line TKI treatment, and its cardiologic and hematologic management. The response of CML to TKI was evaluated according to European LeukemiaNet recommendations [17]. Molecular response (MR) was defined as the finding of detectable BCR-ABL transcripts evaluated by quantitative reverse transcription-polymerase chain reaction with a sensitivity of 3 logs (MR3) or deeper (MR4) [18].

### 2.2. Sample Preparation and Gas Chromatography-Mass Spectrometry (GC-MS) Analysis

The plasma samples from all patients were collected and frozen at −80 °C before being analyzed using a metabolomic tool. The preparation procedure followed a modified version of that reported by Dunn et al. [19]. The plasma samples were slowly thawed in ice and an aliquot of 400 μL was transferred into Eppendorf tubes, then treated with cold methanol 1200 μL, vortex mixed, and centrifuged for 15 min (14,000 rpm, 16.9 G). A supernatant volume of 400 μL was transferred into glass vials; subsequently, it was evaporated overnight to dryness in an Eppendorf vacuum centrifuge. To each vial, a 50 μL volume of 0.24 M (20 mg/mL) solution of methoxylamine hydrochloride in pyridine was added, then the samples were vortex mixed and left to react for 17 h at room temperature. Thereafter, 50 μL of N-methyl-N-trimethylsilyltrifluoroacetamide was added, and the samples were left to react for 1 h at room temperature. Finally, just before gas chromatography-mass spectrometry (GC-MS) analysis, the derivatized samples were diluted with 100 μL hexane and added with 0.01 mg/mL tetracosane as the internal standard.

### 2.3. GC-MS Analysis

All samples were analyzed following a previously reported method of our group [12].

Through this approach, 113 compounds were accurately identified, whereas 28 other metabolites were tentatively assigned relying on the Golm Metabolome Database (GMD) and National Institute of Standards and Technology mass spectral database (NIST libraries). Automated Mass Spectral Deconvolution and Identification System (AMDIS) analysis produced an Excel data sheet that was successively subjected to chemometric analysis.

### 2.4. Statistical analysis

The cumulative incidence of CV-AEs was evaluated using the Kaplan–Meier method after starting TKI treatment. To compare two or more groups of stratified patients, we used the log-rank test. A *p*-value < 0.05 was considered statistically significant. Data analysis was carried out using the statistical package SPSS (version 21; SPSS, Chicago, IL, USA).

An orthogonal partial least-square discriminant analysis (OPLS-DA) was built using SIMCA p+ 13 software (Umetrics, Umea, Sweden). Pareto scaling preceded data analysis. The generated R^2^ and Q^2^ values described the predictive ability and the reliability of the fitting, respectively. A cross-validation analysis of variance was then applied in order to evaluate the significance of the model. The OPLS-DA-derived discriminant metabolites were considered responsible for the differences among groups when their score resulted > 1. 

Subsequently, since metabolites and biological functions are related, we carried out a pathway analysis to enable a functional interpretation. The resulting “metabolome view” showed all the involved metabolic pathways ordered according to the scores deriving from both the enrichment (y-axis) and the topology (x-axis) analyses. All the *p*-values were adjusted for multiple testing. The aim of pathway analysis was to identify the most affected pathways and to verify whether the involved pathway modification affected CV-AEs.

## 3. Results

Table 1 shows the patients’ characteristics. 

**Figure 1:**
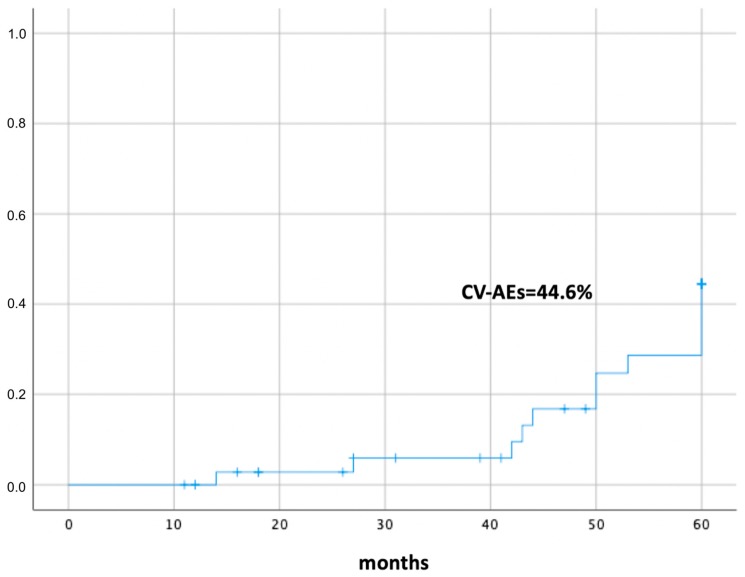
Cumulative incidence of cardiovascular adverse events in 39 patients with chronic myeloid leukemia treated with tyrosine kinase inhibitors. CV-AEs, cardiovascular adverse events.

The mean follow-up time from CML diagnosis was 3.7 years (range 0.9–5 years). A total of 22 patients (56.4%) were treated with first-line TKI therapy, whereas 17 patients (43.6%) underwent second or subsequent TKI treatment lines. The switching was due to inefficacy in 15.3% and intolerance in 28.2%. At the moment of CV-AE development or the last follow-up, 16 patients (41%) were treated with imatinib, 8 (20.5%) with dasatinib, 14 (35.8%) with nilotinib, and 1 with ponatinib. The 60-month cumulative CV-AE incidence was 44.6% ± 9.7% (Figure 1). 

**Figure 2:**
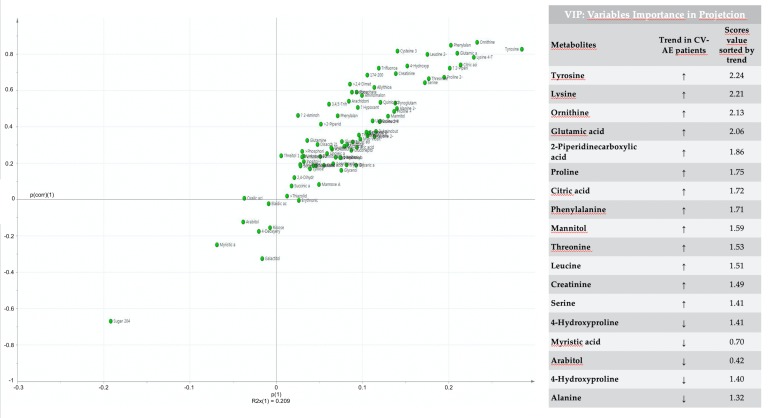
The loadings plot combines the modeled covariance and correlation from the multivariate model (OPLS-DA), the p (1)-axis describe the variable magnitude of each variable, while the p(corr) (1)-axis represents the reliability of each variable. The most important biomarkers, variables importance in projections (VIP), have high magnitude and high reliability and are circled in the figure and listed as metabolites accumulated or depleted in CV-AE patients sorted by trend.

According to the SCORE risk assessment, 72% of the patients showed a low–intermediate risk (SCORE ≤ 5%) and 28% a high–very high risk (SCORE > 5%). Five patients (12.8%) had a positive history of CV diseases. The mean time between the start of treatment and the occurrence of a CV-AE was 44.4 months (range 19–60 months). Overall, in our study population, there were 10 CV-AEs, including 1 episode of hypertension, 4 cardiac events (dissecting aneurysm of the aorta, ST-segment elevation myocardial infarction, reduction of cardiac ejection fraction and atrial fibrillation), 5 pleural or pericardial effusions. Seven CV-AEs were graded as 3 according to the Common Toxicity Criteria, and only one patient died. After the development of CV-AEs, eight patients continued TKI therapy without dose modification, one patient received a reduced dose, and seven patients interrupted the treatment. Several patients underwent additional diagnostic tests such as electrocardiography, cardiac ultrasound, computed tomography angiography, and chest radiography. One patient needed coronarography and invasive procedures such as percutaneous transluminal angioplasty with coronary stenting. The majority of the patients required additional medical therapy. We did not find any significant difference in metabolic profile, according to different TKI (first versus further TKI generations). In multivariate statistical analysis, the samples were clearly separated into two groups, indicating that patients with CV-AEs presented a markedly distinct metabolic profile from that of patients without CV-AEs. The parameters of the model were R^2^Y = 0.76 and Q^2^ = 0.44. A permutation, which showed statistically significant results (*p* = 0.002), was performed to validate the OPLS-DA model. The main discriminant metabolites were tyrosine, lysine, ornithine, glutamic acid, 2-piperdinecarboxylic acid, proline, citric acid, phenylalanine, mannitol, threonine, leucine, creatine, serine, 4-hydroxyproline, and alanine, which were more represented in the CV-AE group. Conversely, myristic acid, arabitol, oxalic acid, and 4-deoxyrithronic acid were less abundant in the CV-AE group (Figure 2). 

Considering each group independently, the pathway analysis identified several shared metabolic ways, as follows: (a) transfer of acetyl groups into mitochondria, (b) amino sugar metabolism, (c) glycerolipid metabolism, and (d) glycine and serine metabolism. Conversely, some pathways were characteristic of only patients with CV-AEs, such as (a) tyrosine and methionine metabolism, (b) sphingolipid metabolism, (c) tryptophan metabolism, (d) citric acid cycle, (e) beta-alanine metabolism, and (f) glutathione metabolism (Table 2).

## 4. Discussion

CV-AEs have become an important public health problem owing to new anticancer TKIs that have “off-target” toxicity. These TKIs block the kinase pathway, including vascular endothelial growth factors, which are involved in the impairment of nitric oxide production and, subsequently, in endothelial dysfunction [20]. Patients with preexisting CV risk factors have a higher risk of developing CV-AEs, regardless of the specific TKI used [21,22]; however, no biomarker has been clearly associated with CV-AEs in CML patients treated with TKIs.

In our study, we used a metabolomic analysis to identify the metabolic fingerprint of CML patients treated with TKIs who subsequently developed CV-AEs. Metabolomic analysis aims to measure the patient dynamic metabolic response to a biological stimulus and has been shown to be a useful tool in the determination of disease biomarkers, identification of pathogenesis, and treatment pathways of diseases [15].

Our GC-MS approach has been confirmed to have better stability and repeatability than other metabolomic techniques, with the key advantage of the use of a number of public and commercialized available databases, such as NIST, which is important for a precise and correct GC-MS peak identification. As shown in Figure 2, the metabolic fingerprint of CML patients with CV-AEs was different from that of CML patients without CV-AEs. Moreover, an altered homeostasis was identified in both groups, as indicated by the following shared pathways (Table 2a,b): (a) transfer of acetyl groups into mitochondria, (b) amino sugar metabolism, (c) glycerolipid metabolism, and (d) glycine and serine metabolism. An impairment of these pathways could possibly be considered as a patient’s response to TKI treatment. Indeed, a previous study reported that TKI were able to reduce phosphorylated hexoses in the leukocytes of CML patients. Moreover, in the leukocytes of newly identified CML patients, most amino acids increased in comparison with controls, whereas they were mostly reduced after TKI treatment [14].

On the contrary, some pathways were exclusive to patients with CV-AEs, such as (a) tyrosine and methionine metabolism, (b) sphingolipid metabolism, (c) tryptophan metabolism, (d) citric acid cycle, (e) beta-alanine metabolism, and (f) glutathione metabolism.

Recently, it has been evidenced that amino acid metabolism is critical in regulating and maintaining vascular functions like vascular tone, coagulation and fibrinolysis, cell growth and differentiation, inflammatory response, and redox homeostasis [20].

Interestingly, in the group of patients with CV-AEs, we found higher serum levels of tyrosine (Figure 2 and Table 2b). Previous data suggested that systemic inflammation may be directly related to tyrosine production. Moreover, in chronic models, the tyrosine level correlated with metabolic, vascular insulin, and erythropoietin resistance [21]. In murine models fed with tyrosine, accumulation of tyrosine in the vascular wall was found to correlate with impaired insulin-induced endothelial relaxation by interfering with the production of the vasodilator nitric oxide [22]. Patients with chronic kidney disease and type 2 diabetes have an increased tyrosine burden, which may contribute to the development of vascular complications [22,23].

Moreover, tyrosine phosphorylation seems to play a crucial role in the regulation of endothelial intercellular adhesion, specifically through the modulation of adherence junctions [24]. The activation of proinflammatory pathways produces an impairment of endothelial barrier function, promoting the disengagement of adherence junctions and favoring the passage of fluids and leukocytes [24].

Notably, only the CML group without CV-AEs presented the alpha-linolenic acid/linoleic acid and cysteine metabolism pathways, which may be associated with a protective effect on the CV system (Table 2). Many studies support the possible vascular protection of seafood omega-3 fatty acids, particularly eicosapentaenoic acid and docosahexaenoic acid, on coronary artery disease. Observational studies have shown that higher levels of the plant-derived omega-3 fatty acid α-linolenic acid was correlated with a moderately lower risk of CVD [25]. Furthermore, a recent study has shown that oral administration of N-acetyl cysteine (NAC) has a strong potential for protecting the diabetic heart at risk of myocardial ischemia through the inhibition of oxidative stress. The administration of NAC results in an increased plasma cysteine level, which enhances the endogenous biosynthesis of glutathione and prevents oxidative stress-induced damage in the cardiomyocytes of diabetic rats [26].

Overall, investigation of the metabolic profiles of CML patients undergoing TKI treatment could provide new insights into biomarkers potentially associated with the risk of TKI-mediated CV complications. Patients with a high risk for CV complications and a deep MR could benefit from a program of TKI dose de-escalation or suspension [27,28].

## 5. Conclusions

Some limitations of our study need to be reported. The first one is that the cohort of patients was rather small and a longitudinal metabolomic profile evaluation from diagnosis is lacking. Furthermore, we did not compare the CML samples with those from patients with CV-AEs not treated with TKI. Nevertheless, the present study is the first to analyze the metabolic fingerprint of CML patients who developed CV-AEs.

This exploratory study showed that CML patients with CV-AEs after TKI treatment have a different metabolic profile, suggesting probable pathways of endothelial damage mediated by the increase of specific metabolites. Tyrosine, which was highly expressed in CML patients with CV-AEs in this study, seems to be a convincing marker of oxidative stress in several acute and chronic diseases. Metabolomic research has a great potential in translating the metabolic fingerprint into personalized therapeutic approaches. These preliminary data should be confirmed in prospective clinical trials.

## Figures and Tables

**Table 1:** Characteristics and cardiovascular profile of 39 patients with chronic myeloid leukemia. BMI, body mass index; CV, cardiovascular; CVD, cardiovascular disease; CV-AEs, cardiovascular adverse events; MR4, molecular response 4; TKIs, tyrosine kinase inhibitors.

Sex, *n* (%)			Response to treatment, *n* (%)		
Male	29	(74.4)	MR4	20	(51.3)
Female	10	(25.6)	No MR4	19	(48.7)
**Age at diagnosis**, mean years (range)	49	(24–70)	**CVD risk factors**, *n* (%)		
**Median follow-up**, mean years (range)	3.7	(0.9–5)	Hypertension	7	(17.9)
**Leukocyte** 10^3^/L, mean value (range)	124	(6.6–300)	Dyslipidemia	10	(25.6)
**Hemoglobin** g/dL, mean value (range)	11.6	(6.4–14.7)	Obesity (BMI > 24.5 kg/m^2^)	9	(23.1)
**Platelet** 10^3^/L, mean value (range)	502	(76–2059)	Severe renal insufficiency	0	(0)
**Splenomegaly**, *n* (%)	14	(36)	Diabetes	7	(17.9)
**Sokal score**, *n* (%)			High CV risk score	11	(28.2)
Low	27	(69)	Low CV risk score	28	(71.8)
Intermediate	8	(21)	**CVD conditions before TKIs**, *n* (%)		
High	4	(10)	Hypertension	2	(5.1)
**Type of TKI**, *n* (%)			Myocardial infarction/angina	1	(2.6)
Imatinib	16	(41)	Arrhythmia	2	(5.1)
Dasatinb	8	(20.5)	**CVD events after TKIs**, *n* (%)		
Nilotinib	14	(35.9)	Pleural or pericardial effusions	5	(12.8)
Ponatinib	1	(2.6)	Hypertension	1	(2.6)
**Line of treatment**, *n* (%)			Atrial fibrillation	1	(2.6)
First	22	(56.4)	ST-elevation myocardial infarction	1	(2.6)
Second	13	(33.4)	Reduction of cardiac ejection fraction	1	(2.6)
Third	2	(5.1)	Dissecting aneurysm of the aorta	1	(2.6)
Fourth	2	(5.1)	**Metabolic effects after TKIs**, *n* (%)		
**Reason for switching**, *n* (%)			Hypercholesterolemia	7	(17.9)
Inefficacy	6	(15.3)			
Intolerance	11	(28.2)			

**Table 2:** Graphic synopsis of metabolic pathways of patients (a) without CV-AEs, and (b) with CV-AEs.

a. Metabolic Pathways of Patients without CV-AEs	Total	Statistics
Transfer of acetyl groups into mitochondria	22	1.4285 × 10 ^−31^
Amino sugar metabolism	33	1.1458 × 10 ^−31^
Glycine and serine metabolism	59	1.5881 × 10 ^−31^
Glycerolipid metabolism	25	6.7412 × 10 ^−32^
Purine metabolism	74	1.3050 × 10 ^−31^
Fructose and mannose degradation	32	3.8378 × 10 ^−32^
Warburg effect	58	1.4761 × 10 ^−31^
Ammonia recycling	32	1.8447 × 10 ^−31^
Cysteine metabolism	26	1.9269 × 10 ^−31^
Alpha-linolenic acid and linoleic acid metabolism	19	1.0902 × 10 ^−31^
**b. Metabolic pathways of patients with CV-AEs**		
Transfer of acetyl groups into mitochondria	22	9.8105 × 10 ^−32^
Amino sugar metabolism	33	1.0445 × 10 ^−31^
Glycine and serine metabolism	59	7.5674 × 10 ^−32^
Glycerolipid metabolism	25	4.1298 × 10 ^−32^
Sphingolipid metabolism	40	1.6407 × 10 ^−31^
Tryptophan metabolism	60	6.8071 × 10 ^−31^
Citric acid cycle	32	9.6546 × 10 ^−32^
Beta-alanine metabolism	34	2.7986 × 10 ^−32^
Glutathione metabolism	21	1.5996 × 10 ^−31^
Tyrosine and methionine metabolism	43	1.2771 × 10 ^−32^

Results are listed indicating the most important pathways, the total number of metabolites identified during the analysis, and the corresponding p-value. The colors indicate unique and shared pathways as follows: light orange specify the group of patients without CV-AEs, light yellow the group of patients with CV-AEs, in orange are displayed pathways shared by both groups (with and without CV-AEs).
